# Response to trametinib, hydroxychloroquine, and bevacizumab in a young woman with *NRAS*-mutated metastatic intrahepatic cholangiocarcinoma: a case report

**DOI:** 10.37349/etat.2024.00246

**Published:** 2024-06-28

**Authors:** Aram A. Musaelyan, Ekaterina M. Anokhina, Alina I. Turdubaeva, Natalia V. Mitiushkina, Anastasia N. Ershova, Anna D. Shestakova, Aigul R. Venina, Evgeny N. Imyanitov, Sergey V. Orlov

**Affiliations:** Humanitas University, Humanitas Research Hospital, Italy; ^1^Department of clinical oncology, Pavlov First Saint Petersburg State Medical University, 197022 Saint Petersburg, Russia; ^2^Department of Antitumor Drug Therapy, St. Luke Clinical Hospital, 194044 Saint Petersburg, Russia; ^3^Department of Tumor Growth Biology, N.N. Petrov National Medical Research Center of Oncology, Ministry of Public Health of the Russian Federation, 197758 Saint Petersburg, Russia; ^4^Department of Medical Genetics, St.-Petersburg Pediatric Medical University, 194100 Saint Petersburg, Russia; ^5^Department of Medical Primatology, National Research Center “Kurchatov Institute”, 354376 Sochi, Russia

**Keywords:** Intrahepatic cholangiocarcinoma, *NRAS* mutation, trametinib, hydroxychloroquine, bevacizumab

## Abstract

Systemic chemotherapy is the main treatment option for patients with advanced intrahepatic cholangiocarcinoma (iCCA), however, its efficacy is limited. Herein, we report a young patient with *NRAS*-mutated chemoresistant metastatic iCCA, who received second-line therapy with a combination of trametinib (MEK1/2 inhibitor), hydroxychloroquine (autophagy inhibitor), and bevacizumab (angiogenesis inhibitor). A significant response was achieved during therapy, resulting in a 25% decrease in the size of tumor lesions after 2 months of treatment and an improvement in the patient’s condition. The duration of this response was 4 months, but the patient died 10 months after the initiation of this triple therapy. This case report and the analysis of other available studies warrant further investigations on combined MEK and autophagy inhibition in *RAS*-mutated tumors.

## Introduction

Cholangiocarcinoma (CCA) is an uncommon type of hepatobiliary cancer, accounting for approximately 3% of gastrointestinal malignancies [1]. CCAs originate from the epithelium of the bile duct and are classified into intrahepatic [intrahepatic CCA (iCCA)], perihilar, and distal carcinomas [2]. These types of CCAs are distinct in terms of epidemiology, clinical presentation as well as morphological and molecular characteristics [3, 4].

iCCA is a highly aggressive cancer, which accounts for approximately 10% of CCA cases [5]. Meanwhile, the incidence of iCCA has been steadily increasing over the past few decades [6]. iCCA is anatomically located between bile ductules and second-order bile ducts [6]. Thus, the early stages of the disease are usually asymptomatic and often diagnosed due to chance [7]. The potentially curative option for localized iCCA is a surgical resection followed by the subsequent use of adjuvant capecitabine. However, the survival outcomes for early-stage iCCA are modest, with a median overall survival (OS) of less than 5 years [8, 9].

Approximately 70–80% of iCCA patients are not eligible for curative surgery at the time of the initial disease diagnosis [10]. Systemic therapy is the main treatment choice for subjects with advanced iCCA [5]. Until recently, the first-line therapy for metastatic iCCA relied on a combination of gemcitabine and cisplatin, however, this treatment produced OS of less than one year [6, 11]. The current standard of care involves adding durvalumab to this drug combination, as the relevant phase III clinical trial has shown an improvement in OS from 11.5 months to 12.8 months [12, 13]. There are no options rendering significant clinical benefit after the failure of first-line therapy [6].

iCCAs often carry driver genetic mutations, particularly *FGFR2* fusions or mutations, *HER2* amplification, *BRAF* V600E substitutions, and hotspot alterations affecting *IDH1*, *KRAS*, and *NRAS* genes [14]. While activating events involving *FGFR2*, *IDH1*, *HER2*, and *BRAF* oncogenes are druggable, targeting *RAS*-mutated tumors remains a challenge [15]. *RAS* mutations result in the activation of MEK kinase, however, clinical trials utilizing single-agent MEK inhibitors for this type of cancer have failed [16, 17]. Preclinical studies suggest that the escape from MEK down-regulation may be due to autophagy activation [18]. As a result, several case studies demonstrated that combined inhibition of MEK kinase and autophagy may result in the shrinkage of *RAS*-mutated tumors, although negative results have been published as well [18–20]. This report describes a young woman with metastatic *NRAS*-positive iCCA, who had a remarkable response to a combined second-line therapy with MEK1/2 inhibitor trametinib, autophagy inhibitor hydroxychloroquine, and anti-angiogenic antibody bevacizumab.

## Case report

A 22-year-old woman was admitted to the hospital in October 2022 with complaints of nausea and vomiting up to 3–4 times per day. The patient had no previous medical or surgical history. She had no history of smoking or alcohol abuse and was not taking any medications. The imaging procedures were conducted on October 14th, 2022. An abdominal computed tomography (CT) showed multiple confluent heterogeneous lesions in both lobes of the liver, some of which had a necrotic component. A conglomerate measuring 100 mm × 76 mm was observed in segment 4 (S4), spreading to S8 and S2, involving the capsule, the left and middle hepatic veins, and some branches of the portal vein. There was a presence of ascites and a conglomerate of lymph nodes (74 mm × 35 mm) along the splenic and common hepatic arteries, extending to the tail of the pancreas. A chest CT scan revealed multiple round metastatic lesions in all lobes of both lungs. The largest lesion (16 mm × 15 mm) was observed in the S1 + 2 of the left lung. Additionally, there were supradiaphragmatic lymph nodes with altered structure measuring up to 36 mm ×16 mm, paratracheal and bifurcation lymph nodes measuring up to 14 mm along the short axis, and left supraclavicular lymph nodes measuring up to 9 mm × 7 mm. A pelvic magnetic resonance imaging revealed a significant amount of fluid in the pelvis.

**Figure 1:**
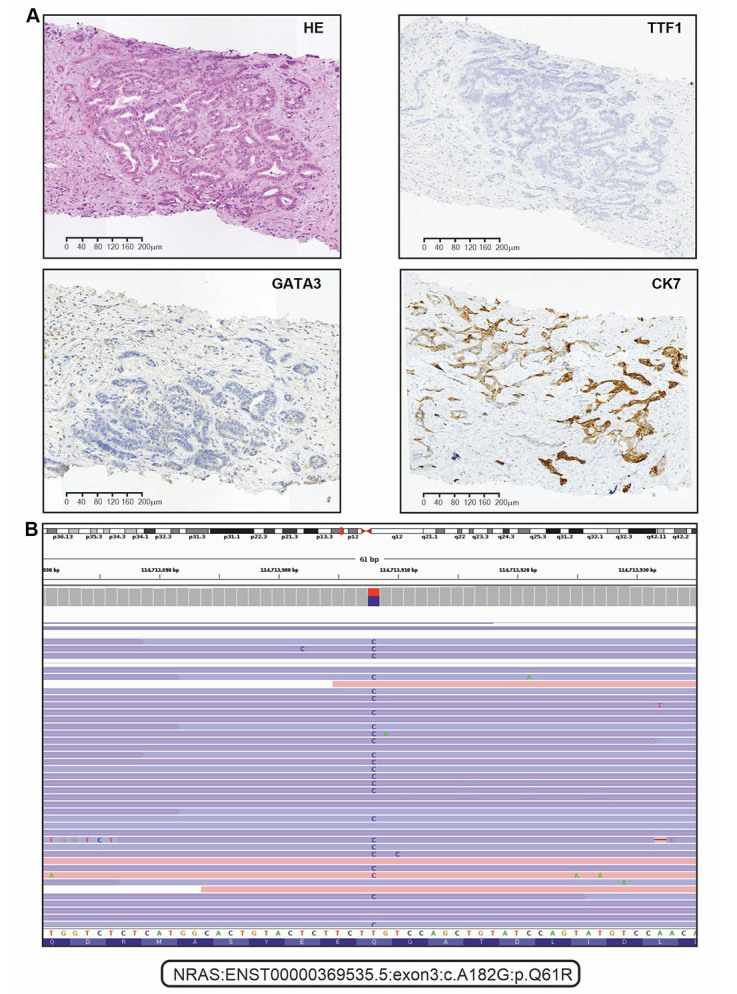
Immunohistochemical (IHC) and next-generation sequencing analysis of tumor tissue. (A) Representative microphotographs for hematoxylin and eosin (HE) staining and selected examples of IHC analysis (positive staining for CK7, and negative staining for TTF1 and GATA3); (B) the alignments of sequencing reads at the position of the *NRAS* p.Q61R mutation as shown in the IGV browser (version 2.16.1)

The patient underwent an ultrasound-guided liver biopsy on October 17th, 2022. Immunohistochemical analysis showed that the tumor sample was positive for CK7, but negative for CDX-2, CK20, GATA3, PAX8, and TTF1 (Figure 1A). Consequently, this malignancy was classified as a well-differentiated iCCA (large duct type).

The analysis of *FGFR1-3*, *KRAS*, *NRAS*, *BRAF*, *HER2*, and *IDH1* genes was done with custom next-generation RNA sequencing [14]. Receptor tyrosine kinase rearrangements (*RET* and *NTRK1–3*) were analyzed by polymerase chain reaction (PCR) tests [21, 22]. Microsatellite instability (MSI status) was determined by a standard pentaplex panel (BAT25, BAT26, NR21, NR22, and NR24 mononucleotide markers) [23]. Molecular analysis revealed that the tumor was microsatellite stable and wild-type for *NTRK, BRAF, IDH1, RET*, *HER2*, *FGFR1-3*, and *KRAS* genes. However, *NRAS* p.Q61R mutation was identified (Figure 1B).

The patient was started on systemic therapy using a combination of gemcitabine and cisplatin on November 1st, 2022. She received gemcitabine at a dose of 1000 mg/m^2^ and cisplatin at a dose of 25 mg/m^2^ via intravenous administration on the first and eighth days of a three-week treatment cycle. On December 14th, 2022, a contrast-enhanced CT scan of the chest, abdomen, and pelvis revealed progressive disease according to Response Evaluation Criteria in Solid Tumors version 1.1 (RECIST 1.1). The scan showed an increase in the size of lesions in the liver, the appearance of new interhepatic lumps, increased size and number of metastases in the lungs, and enlargement of intrathoracic lymph nodes.

**Figure 2:**
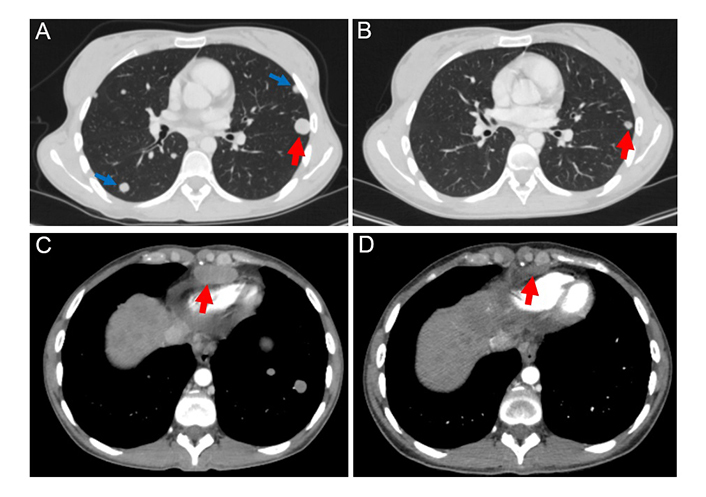
The target lesion located in the S1 + 2 of the left lung (marked by red arrows) decreased in size by 50% after 2 months of therapy with trametinib, hydroxychloroquine, and bevacizumab [from 18 mm (A) to 9 mm (B)]. Non-target lesions (marked by blue arrows) were detectable at baseline (A), but absent in the follow-up scan (B). The examination of the supradiaphragmatic lymph nodes showed their reduction from 18 mm (C) to 9 mm (D) during the therapy (a decrease of 50%)

**Figure 3:**
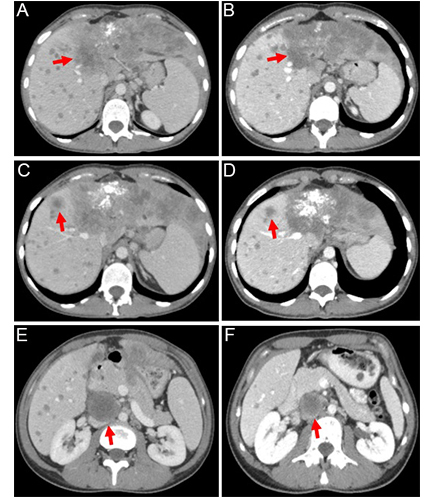
Contrast-enhanced abdominal computed tomography (CT) scans were performed at baseline (left) and after 2 months of the triple-agent therapy (right). The treatment led to a reduction in the size of the following lesions (marked by red arrows): the tumor located in S4 of the liver [from 147 mm (A) to 115 mm (B); a decrease of 22%], the lesion in S8 of the liver [from 20 mm (C) to 17 mm (D); a decrease of 15%], and the conglomerate between the head of the pancreas and the inferior vena cava [from 42 mm (E) to 35 mm (F); a decrease of 17%]

The patient refused to receive the FOLFOX regimen (oxaliplatin, 5-fluorouracil, and folinic acid), which is a standard second-line treatment for this disease. Considering the failure of the first-line platinum-containing chemotherapy, the highly aggressive nature of the tumor, and the presence of an *NRAS* p.Q61R mutation, the patient was offered an experimental treatment. This therapy included trametinib (2 mg once daily), hydroxychloroquine (400 mg twice daily), and bevacizumab (7.5 mg/m^2^ every 3 weeks). She provided informed consent and started this therapy on December 30th, 2022. The treatment resulted in a self-reported rapid improvement in the general condition of the patient. On February 21st, 2023, a follow-up CT revealed a decrease in the number and size of metastatic lesions in the lungs, particularly a reduction of the lesion located at the S1 + 2 of the left lung [from 18 mm × 17 mm (Figure 2A) to 9 mm × 8 mm (Figure 2B)]. The size of the supradiaphragmatic lymph node decreased from 39 mm × 18 mm (Figure 2C) to 20 mm × 9 mm (Figure 2D). Tumor shrinkage was also observed in the liver: the lesion in S4, spreading to S8 and S2, was reduced in size from 147 mm × 89 mm (Figure 3A) to 115 mm × 65 mm (Figure 3B), and the lump located in S8 shrank from 20 mm × 20 mm (Figure 3C) to 17 mm × 15 mm (Figure 3D). There was also a reduction of the conglomerate located between the head of the pancreas and the inferior vena cava from 42 mm × 40 mm (Figure 3E) to 35 mm × 20 mm (Figure 3F). At the same time, the size of lymph node lesions along the splenic and common hepatic arteries did not change. The treatment was accompanied by a partial resolution of ascites. Altogether, there was a 25% decrease in the sum of diameters of target lesions, which was defined as a stable disease according to RECIST 1.1.

On April 26th, 2023, a follow-up contrast-enhanced CT scan of the chest, abdomen, and pelvis revealed a stabilization in the size of the target lesions. However, there was a notable increase in the size of non-target lesions in the lungs and liver compared to the previous CT images. Also, new lesions, measuring up to 3 mm, appeared in the lungs. Thus, the duration of response to the combination therapy of trametinib, hydroxychloroquine, and bevacizumab was 4 months. During therapy, only a grade 2 papulopustular rash appeared in January 2023, which was relieved with symptomatic therapy.

It was suggested that the patient continue therapy with the addition of pembrolizumab 200 mg every three weeks. However, a disease progression was observed after 4 cycles of this treatment. Subsequently, the patient did not receive any further antitumor therapy. The patient died in October 2023. The OS since the initiation of experimental therapy was 10 months.

## Discussion

This article describes a successful second-line treatment of *NRAS*-mutated chemo-resistant metastatic iCCA using a combination of trametinib, hydroxychloroquine, and bevacizumab.

The combination of gemcitabine and cisplatin, along with anti-programmed cell death protein 1 (anti-PD-1, pembrolizumab)/PD-1 ligand (PD-L1, durvalumab) therapy, is currently being used as the first-line treatment for patients with advanced iCCA [12, 24]. However, this patient started the treatment with conventional chemotherapy. This was due to the lack of local approval of immunotherapy for this cancer type at the time of treatment decision, although a few months later immune checkpoint inhibitors were incorporated in the national standards of iCCA treatment.

Based on a single prospective randomized phase III trial, patients with advanced biliary tract cancers experiencing progression on the first-line chemotherapy are usually offered the FOLFOX regimen [25]. This treatment option has shown a modest, but statistically significant improvement in survival compared to a placebo [25]. For patients with iCCA, the median progression-free survival (PFS) and OS were 3.3 months and 5.7 months, respectively [25]. Targeted therapy demonstrated a promise only for certain molecular subtypes of iCCA [6].

Targeting oncogenic RAS proteins remains a challenge, with only *KRAS* G12C substitution being currently accessible for therapeutic inhibition [19]. RAS family proteins play a crucial role in the mitogen-activated protein kinase (MAPK) signaling pathway. Therefore, targeting downstream proteins of the cascade is a promising option. Several MEK inhibitors are available for clinical use, e.g., cobimetinib, trametinib, selumetinib, and binimetinib. However, these drugs have limited efficacy in *RAS*-mutated tumors [19]. Autophagy has been shown to contribute to the escape from MEK-targeted therapy [16]. Therefore, a combination of a MEK inhibitor and an autophagy inhibitor, such as hydroxychloroquine, is a promising option [16]. While this combination has proven effective in preclinical experiments [26, 27], it demonstrated generally modest efficacy in clinical investigations (Table 1). However, no clinical studies, with the exception of one case report, utilized bevacizumab [28]. Bevacizumab is believed to improve the delivery of anticancer drugs to cancer cells and may have a direct antitumor activity in some circumstances [29, 30, 31]. This report and a previous case observation suggest that the addition of bevacizumab may be essential for combined MEK and autophagy inhibition [28]. The triplet combination of MEK antagonist, hydroxychloroquine, and bevacizumab deserves further evaluation in patients with *RAS*-mutated tumors.

**Table 1:** Overview of studies investigating the efficacy of using MEK inhibitors combined with hydroxychloroquine in pre-treated patients

**Tumor type**	**Study design**	**Number**	**MEK inhibitor**	**Endpoints**	**Reference**
Biliary tract cancer	Phase II trial	2	Trametinib	PFS: 2.48 months; OS: 3.1 months.	[32]
Colorectal cancer	Case report	1	Binimetinib (+ bevacizumab)	Best response: stable disease; Duration: approximately 4 months.	[28]
Pancreatic cancer	Case report	1	Trametinib	Best response: partial response; Duration: approximately 4 months.	[26]
Pancreatic cancer	Case report	2	Trametinib	Best response: stable disease; Duration: approximately 4 months and 7 months.	[33]
Pancreatic cancer	Case report	1	Trametinib	Best response: stable disease; Duration: approximately 7 months.	[34]
Pancreatic cancer	Case report	1	Trametinib	Best response: progressive disease; Duration: approximately 2 months.	[35]
Pancreatic cancer	Retrospective	9	Trametinib	DCR: 63%; PFS: 5.7 months; OS: 6.6 months.	[20]
		1	Cobimetinib		
Pancreatic cancer	Retrospective	8	Trametinib	Best response: stable disease (16.7%); PFS: 2.0 months; OS: 4.2 months.	[16]
NSCLC	Phase II trial	9	Binimetinib	Best response: stable disease (11.1%); PFS: 1.9 months; OS: 5.3 months.	[18]

NSCLC: non-small cell lung cancer; DCR: disease control rate; PFS: progression-free survival; OS: overall survival

In conclusion, the combination of trametinib, hydroxychloroquine, and bevacizumab has shown efficacy in second-line therapy for a patient with metastatic *NRAS*-mutated iCCA. This observation warrants a further prospective clinical trial.

## Abbreviations

CT: computed tomography
iCCA: intrahepatic cholangiocarcinoma
OS: overall survival

## Declarations

### Availability of data and materials

The raw data supporting the conclusions of this manuscript will be made available by the authors, without undue reservation, to any qualified researcher.

### Copyright

© The Author(s) 2024.
